# Facial esthetic outcome of functional followed by fixed orthodontic treatment of class II division 1 patients

**DOI:** 10.1186/s40510-019-0294-9

**Published:** 2019-11-25

**Authors:** Mary-Eleni Zouloumi, Kleopatra Tsiouli, Simeon Psomiadis, Olga-Elpis Kolokitha, Nikolaos Topouzelis, Nikolaos Gkantidis

**Affiliations:** 1 Faculty of Dentistry, Aristotle University of Thessaloniki, University Campus, 54124 Thessaloniki, Greece; 2 Private Practice, Gr. Marasli 36, 49132 Corfu, Greece; 3 Department of Oral and Maxillofacial Surgery, Dental School, National and Kapodistrian University of Athens, Thivon 2, Goudi, 11527 Athens, Greece; 4 Department of Orthodontics, Faculty of Dentistry, Aristotle University of Thessaloniki, University Campus, 54124 Thessaloniki, Greece; 5 Department of Orthodontics and Dentofacial Orthopedics, University of Bern, Freiburgstrasse 7, 3010 Bern, Switzerland

**Keywords:** Outcome assessment, Facial esthetics, Functional orthodontic appliances, Class II, division 1

## Abstract

**Objectives:**

To assess the perceived facial changes in class II division 1, convex profile patients treated with functional followed by fixed orthodontic appliances.

**Subjects and methods:**

The study sample consisted of 36 pairs of pre- and post-treatment photographs (frontal and profile, at rest) of 12 patients treated with activator, 12 with twin-block, and 12 controls with normal profiles, treated without functional appliances. All photographs were presented in pairs to 10 orthodontists, 10 patients, 10 parents, and 10 laypersons. Visual analog scale (VAS) ratings of changes in facial appearance were assessed.

**Results:**

The patient groups were similar in sex distributions, age, and treatment duration. The different rater groups showed strong to excellent agreement. There were no significant differences among treatment groups (*F* = 0.91; *P* = 0.526; Wilks lambda = 0.93), raters (*F* = 1.68; *P* = 0.054; Wilks lambda = 0.83), and when testing the combined effect of treatment and rater on the results (*F* = 0.72; *P* = 0.866; Wilks lambda = 0.85). The raters detected slightly more positive changes in the activator and twin-block groups, compared to the control group, regarding the lower face and the lips, but these findings did not reach significance. Furthermore, their magnitude hardly exceeded 1/20th of the total VAS length.

**Limitations:**

Retrospective study design.

**Conclusions:**

The perceived facial changes of convex profile patients treated with functional, followed by fixed orthodontic appliances, did not differ from those observed in normal profile patients, when full-face frontal and profile photos were simultaneously assessed. Consequently, professionals should be skeptical regarding the improvement of a patient’s facial appearance when this treatment option is used.

## Introduction

Facial esthetics play a significant role in everyday life and interpersonal relationships [1]. Orthognathic and orthodontic irregularities are frequently accompanied by suboptimal facial esthetics. This includes class II malocclusions that have convex profiles and retruded mandibular position of hard and soft tissues [2–4].

During active growth, class II patients can be treated effectively with functional orthopedic appliances, where orthodontists attempt to modify the skeletal growth [5]. Activator and twin-block are two commonly used appliances aiming to enhance mandibular growth in patients with convex profiles due to a retrognathic mandible [5, 6]. However, a recent systematic review revealed a relatively small improvement of the facial outline when removable functional appliances were used [5].

Improvement of facial esthetics, including the dental appearance, is the main reason for which patients seek orthodontic treatment [7]. Therefore, patients’ satisfaction is fulfilled when their facial appearance is actually improved and not only when proper dentoskeletal relations are restored, according to objective measurements [8]. Thus, when assessing the orthodontic treatment outcome, which aims to improve facial esthetics, studies need to focus on the opinion of different groups of evaluators, including also the subjective layperson’s opinion, which comprise the target group of our treatments. Previously, only a small favorable change in facial appearance was perceived when raters were asked to evaluate the esthetic outcome of functional orthodontic treatment on convex profile class II division 1 patients [9]. This underlines the need for more studies investigating the perceived improvement of facial appearance, achieved by treatments that have such aims.

Current literature focuses on the improvement of patients’ facial profile following skeletal and dentoalveolar class II correction [2, 3, 5, 10–16]. Indeed, functional appliances may influence to some degree the patients’ facial profile [5, 10, 11, 13, 15] and this might be perceivable by the human eye as more attractive [9, 13, 14]. However, facial attractiveness is evaluated in everyday life from different angles and not only from the profile view and this may impact esthetic assessments [17]. Furthermore, other characteristics, such as hair or skin texture, may also influence the perception of facial profile esthetics [16].

So far, there is only one study that investigated the esthetic improvement of convex profile patients, after treatment with functional followed by fixed orthodontic appliances, using actual images [9]. In that study, raters assessed actual facial profile photographs before and after the orthodontic intervention and perceived a slight improvement in the esthetic appearance. The primary aim of this study was to assess treatment outcomes taking into consideration the total facial appearance as viewed from profile and frontal photographs. Secondarily, possible differences between groups of raters, activator and twin-block appliances, and parts of the face were explored.

## Material and methods

To allow for valid comparisons, the sample was identical and the design similar to that used on a previous study [9]. Data from that study were used to perform a power analysis, which showed that the sample size was adequate [9]. The sample was obtained consecutively, from the most recent patient records of the Department of Orthodontics at the Aristotle University of Thessaloniki that fulfilled the inclusion criteria. Two test groups and one control group, consisting of 12 persons each, were formed. Pre-treatment diagnostic records were used for sample selection. Post-treatment records were only reviewed to confirm availability.

The eligibility criteria for the test groups were (1) full pre- and post-treatment diagnostic records, (2) class II (more than half molar cusp bilaterally) division 1 malocclusion, (3) convex profile defined by facial contour angles (formed by the glabella-subnasale line and the extension of the subnasale-pogonion line) greater than 15° for males and greater than 17° for females on the initial lateral cephalometric radiograph, (4) mixed dentition at start of the orthodontic intervention, (5) complete treatment with activator or twin-block followed by fixed orthodontic appliance treatment, (6) non-extraction treatment, (7) white racial background, and (8) no craniofacial malformations, syndromes, clefts, teeth absences, severe facial asymmetries, or functional mandibular shift over 1 mm [9].

The control group consisted of 12 patients who fulfilled the same criteria as the test groups but differed in the following: (1) class I or class II with less than a half-cusp distal molar relation bilaterally, (2) normal facial contour, and (3) complete treatment with fixed appliances, without the use of any functional orthodontic appliances.

As reported previously [9], the treatment groups were similar in sex distributions, age, and treatment duration. The activator and twin-block groups were also similar in facial convexity and pre-treatment overjet, but they differed significantly with the control group in these parameters. Post-treatment overjet was within normal values in all groups, suggesting successfully treated patients in this aspect. More detailed information on the sample characteristics is available in Additional file 1: Table S1, as well as in a previous publication [9].

The final sample consisted of pre- and post-treatment photographs of 36 patients (18 male and 18 female). During image acquisition, patients were positioned with the Frankfort horizontal plane parallel to the ground, teeth in maximum intercuspation, and lips at rest. All photographs were in digital form and were edited to have a white background and similar brightness and contrast. Any skin imperfections and any jewelry were digitally removed (Adobe Photoshop CS6, Adobe Systems, San Jose, CA, USA). This image processing was made to avoid bias due to factors affecting facial attractiveness which were, however, not related to the testing hypothesis.

The photographs were presented to 120 raters, which formed four different groups: 30 orthodontists (15 male, 15 female), 30 patients (15 male, 15 female), 30 parents of corresponding patients (15 male, 15 female), and 30 laypersons (15 male, 15 female). The patients’ group of evaluators comprised class II division 1 patients who were treated in a local private practice during the study and were between 9 and 16 years of age. The rest of the groups consisted of adults between 20 and 65 years of age. During the distribution of the questionnaires to the parents and laypersons, it was taken into account to include adults of various socioeconomic statuses, educational levels, and fields. All raters had no relation with the patients in the sample, and orthodontists were not involved at their treatment. All raters were randomly selected and were the first 30 of each group that accepted to participate in the study.

**Fig. 1:**
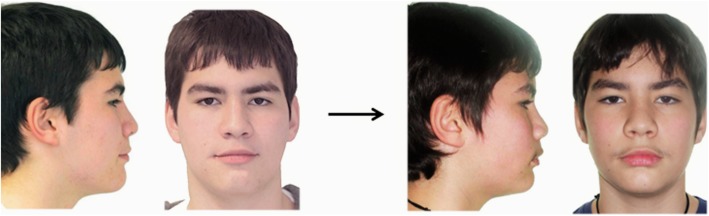
Photographs of a selected patient as presented to raters. The post-treatment photograph is presented to the left and the pre-treatment photograph to the right

All pre- and post-treatment photographs were presented in pairs. They were printed in a A4-size paper page, in landscape orientation, and were arranged in three photo albums (12 patients per album, six males and six females), according to a previously verified setup [9]. Each album consisted of four patients of each treatment approach (four activator, four twin-block, and four controls). This way, 10 assessments of each patient were obtained by each rater group. In each album, half of the patients were presented with the pre-treatment photographs on the left and the post-treatment photographs on the right and half were presented in reverse order. In addition, half of all patients in an album were presented with the profile photograph before the frontal one and half in reverse order. All photographs were aligned based on the lateral canthus of the eyes and were adjusted to be of the same size (Fig. 1).

**Fig. 2:**
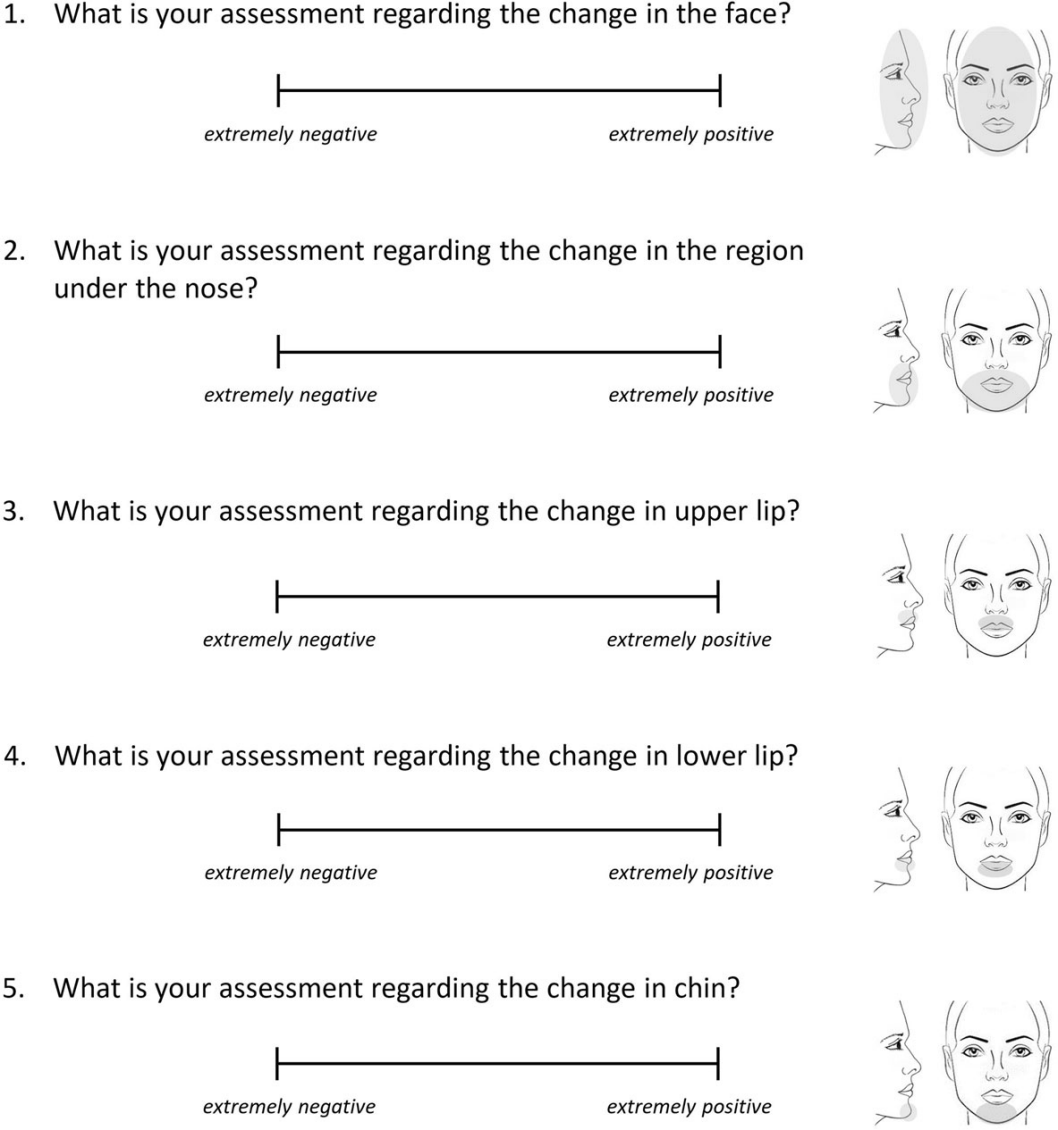
The questionnaire provided to the raters. “Extremely negative” corresponds to 0 and “extremely positive” corresponds to 100 VAS value

The raters were asked to complete a standardized and previously validated questionnaire [9, 18, 19], while looking at the presented album. First, they were asked to provide the following demographic information: gender, date of birth, profession, and education level. Afterwards, for each set of photographs, the raters answered five questions using a visual analog scale (VAS) of 0–100 mm; the left side of the scale was described as extremely negative and the right side was described as extremely positive. Each of the five questions referred to different regions of the face and was accompanied by an illustration to be easily perceived (Fig. 2).

The questionnaires were printed and distributed to all evaluators by a single researcher (M.Z.). The rating of the photographs was conducted in a quiet, nonclinical environment with adequate lighting. The researcher was in the room during the procedure, to answer potential questions, without however interfering in the evaluation. At the beginning, standardized instructions were given regarding the assessment process. Participants were not informed that the photographs showed orthodontic patients before and after treatment to ensure that their judgments were not biased. After viewing each photograph for as long as they considered necessary, raters made a mark on the VAS, according to their perception of facial change. Approximately 12 min were required for a questionnaire to be filled by an evaluator. This time is considered acceptable to avoid fatigue of the raters that might lead to unreliable responses [9].

Ratings were transformed to continuous metric variables for statistical analysis by measuring the distance between the start and the marks on the VAS with a digital caliper. All measurements were recorded in a Microsoft Excel sheet (Microsoft Office 365). In half of the sample, when the post-treatment condition was presented to the left, VAS measurements were adjusted by subtracting each value from 100 to conform with the other half of the ratings.

One month after the measurements of the questionnaires, the same researcher re-measured 30 VAS scores to assess method error. To assess repeatability of ratings, 12 raters (3 orthodontists, 3 patients, 3 parents, 3 laypeople—6 males, 6 females) reassessed the images after a 4-week washout period.

### Statistical analysis

The statistical analysis was carried out by using SPSS software (version 20.0; IBM, Armonk, NY, USA). Levene’s test showed homogeneity of variances in all cases. Data were tested for normality with the Shapiro-Wilk test and were not normally distributed in a few cases. Thus, parametric and nonparametric statistics were applied depending on normality.

Treatment group similarity was tested previously and proved adequate [9].

Intraexaminer agreement on the repeated VAS measurements was tested with the Wilcoxon signed-rank test. Random error was assessed with Dahlberg’s formula.

Intrarater agreement (test-retest reliability) of repeated VAS ratings was tested with the Wilcoxon signed-rank test and the intraclass correlation coefficient (two-way random model, absolute agreement, average measures). A one-sample *t* test was used for testing if the mean differences between the two measurements are statistically different from 0.

Internal consistency for professionals, patients, parents, and laypeople was assessed by the calculation of the Cronbach's alpha for each test group separately. The Cronbach's alpha was based on median scores of the assessors in each group. The effect of deleting each item once from a subscale on the obtained alpha values was also examined. A level above 0.8 was considered high consistency and above 0.7 was considered acceptable.

The interrater agreement among groups was determined by means of intraclass correlation coefficients (two-way mixed model, absolute agreement, average measures). Each patient was rated by 10 members from each rater group; therefore, the median VAS score for each item was used to obtain a more representative approximation of each group’s assessments for the specific patient.

A level above 0.7 was considered strong agreement and moderate agreement was at 0.5 and 0.6. The validation of the specific questionnaire on a similar population has been published previously [9] and was further tested in the present study.

Two-way multivariate analysis of variance was used to evaluate differences among group ratings. The assessment score for each patient was calculated as described above for interrater agreement. Responses to the five items of the questionnaire were the five dependent variables, and the treatment groups (activator, twin-block, control group) and the rater groups (orthodontists, patients, parents, laypeople) were the independent variables. Equality of covariances of the dependent variables was tested with Levene’s test for equality of error variances. Post hoc pairwise comparisons were performed with the Fisher's least significant difference test.

In all cases, a two-sided significance test was carried out at an alpha level of 0.05. The level of significance used for the study was set at 0.05. A Bonferroni correction was applied for pairwise a posteriori multiple comparison tests.

## Results

There was no statistically significant difference between the first and second VAS measurements (intraexaminer error; *P* > 0.05); random error was minimal (0.27 mm).

There was no statistically significant difference between repeated VAS ratings (intrarater agreement; *P* > 0.01) of all 12 raters. There was a strong to almost perfect intrarater agreement for less than half of the cases tested. Moderate to weak agreement was evident for the rest (Table 1). Mean differences between the two repeated ratings performed by 12 raters were minimal. The one-sample *t* test showed that in all cases, mean differences between the repeated ratings were lower than 7 VAS values (7%) and not significantly different from 0. However, the observed variation was high (Table 2).

**Table 1:** Intra-rater agreement of repeated VAS ratings tested through the intraclass correlation coefficient (ICC, two-way random model, absolute agreement, results regarding average measures)

Intra-rater agreement	Orthodontists	Patients	Parents	Laypersons	All
Face	0.61 (CI 0.25, 0.80)	0.74 (CI 0.49, 0.87)	0.78 (CI 0.57, 0.89)	0.40 (CI − 0.18, 0.69)	0.68 (CI 0.56, 0.77)
Lower face	0.45 (CI − 0.08, 0.72)	0.60 (CI 0.21, 0.80)	0.70 (CI 0.41, 0.85)	0.48 (CI − 0.02, 0.73)	0.61 (CI 0.46, 0.72)
Upper lip	0.37 (CI − 0.25, 0.68)	0.80 (CI 0.60, 0.90)	0.69 (CI 0.39, 0.84)	0.55 (CI 0.12, 0.77)	0.68 (CI 0.56, 0.77)
Lower lip	0.58 (CI 0.18, 0.78)	0.23 (CI − 0.51, 0.61)	0.59 (CI 0.19, 0.79)	0.50 (CI 0.05, 0.74)	0.43 (CI 0.21, 0.59)
Chin	0.50 (CI 0.00, 0.74)	0.59 (CI 0.20, 0.79)	0.80 (CI 0.62, 0.90)	0.53 (CI 0.09, 0.76)	0.64 (CI 0.50, 0.74)

**Table 2:** Mean differences between two repeated ratings performed by 12 raters and one-sample *t* test testing if the mean difference is significantly different from 0 (systematic error; *P* < 0.01)

	*t*	df	Sig. (two-tailed)	Mean difference	SD	95% confidence interval of the difference	
						Lower	Upper
All raters							
Face	2.20	143	0.029	3.94	21.42	0.41	7.46
Lower face	0.32	143	0.746	0.67	24.61	− 3.39	4.72
Upper lip	0.55	143	0.584	0.96	20.91	− 2.49	4.40
Lower lip	0.48	143	0.629	1.08	26.73	− 3.32	5.48
Chin	− 0.32	143	0.749	− 0.68	25.38	− 4.86	3.50
Orthodontists							
Face	1.22	35	0.231	3.85	18.97	− 2.57	10.27
Lower face	0.53	35	0.600	1.94	21.97	− 5.50	9.37
Upper lip	0.55	35	0.584	1.87	20.32	− 5.00	8.75
Lower lip	1.31	35	0.200	4.32	19.84	− 2.39	11.04
Chin	0.57	35	0.574	1.99	21.02	− 5.13	9.10
Patients							
Face	0.83	35	0.410	3.39	24.39	− 4.87	11.64
Lower face	− 0.64	35	0.524	− 3.35	31.15	− 13.89	7.20
Upper lip	− 0.75	35	0.458	− 2.94	23.53	− 10.90	5.02
Lower lip	− 0.73	35	0.470	− 4.80	39.44	− 18.15	8.55
Chin	− 1.18	35	0.248	− 6.98	35.58	− 19.01	5.06
Parents							
Face	1.30	35	0.202	4.18	19.30	− 2.35	10.71
Lower face	0.35	35	0.725	1.30	21.94	− 6.13	8.72
Upper lip	0.21	35	0.832	0.69	19.50	− 5.91	7.29
Lower lip	− 0.55	35	0.583	− 2.00	21.65	− 9.32	5.33
Chin	− 0.98	35	0.333	− 3.14	19.20	− 9.63	3.36
Laypersons							
Face	1.11	35	0.274	4.33	23.38	− 3.58	12.24
Lower face	0.73	35	0.469	2.78	22.72	− 4.91	10.46
Upper lip	1.24	35	0.223	4.20	20.30	− 2.67	11.07
Lower lip	2.01	35	0.052	6.79	20.28	− 0.07	13.66
Chin	1.51	35	0.139	5.42	21.49	− 1.85	12.69

The internal consistency of the items of the questionnaire was generally acceptable both within and between groups, with a Cronbach’s alpha value higher than 0.9 in all cases, except from the patient group of raters, where the value was lower, but still above 0.7. The explorative elimination of any item consistently did not increase alpha values significantly in any case. Thus, it was reasonable to keep all items (Table 3).

**Table 3:** Internal consistency of the answers of all rater groups for the three treatment groups, measured by Cronbach’s alpha, and influence of the deletion of each item by each subscale on Cronbach’s alpha values

Group	Items	Cronbach’s alpha			
		Activator (if item deleted)	Twin block (if item deleted)	Control (if item deleted)	All (if item deleted)
Orthodontists	All	0.939	0.948	0.981	0.956
	Face	0.945*	0.931	0.978	0.952
	Lower face	0.905	0.921	0.976	0.933
	Upper lip	0.906	0.944	0.979	0.946
	Lower lip	0.932	0.957*	0.974	0.951
	Chin	0.933	0.928	0.972	0.945
Patients	All	0.770	0.886	0.873	0.855
	Face	0.707	0.830	0.822	0.798
	Lower face	0.826*	0.836	0.842	0.836
	Upper lip	0.627	0.871	0.833	0.810
	Lower lip	0.741	0.891*	0.879*	0.850
	Chin	0.717	0.877	0.845	0.826
Parents	All	0.951	0.953	0.974	0.960
	Face	0.951	0.938	0.962	0.952
	Lower face	0.929	0.933	0.968	0.945
	Upper lip	0.939	0.927	0.966	0.945
	Lower lip	0.930	0.939	0.963	0.944
	Chin	0.952*	0.968*	0.976*	0.963*
Laypersons	All	0.963	0.955	0.956	0.960
	Face	0.959	0.941	0.943	0.952
	Lower face	0.948	0.948	0.931	0.945
	Upper lip	0.954	0.936	0.959*	0.954
	Lower lip	0.955	0.947	0.957*	0.954
	Chin	0.956	0.952	0.933	0.949
All	All	0.936	0.933	0.950	0.941
	Face	0.932	0.911	0.936	0.929
	Lower face	0.922	0.905	0.935	0.923
	Upper lip	0.911	0.916	0.942	0.925
	Lower lip	0.920	0.927	0.942	0.931
	Chin	0.922	0.929	0.938	0.930

*Cases where item deletion resulted in increased Cronbach’s alpha value of the corresponding subscale

The different rater groups showed strong to excellent agreement upon rating of each treatment group in all items, although the confidence intervals for certain cases were wide (Table 4).

**Table 4:** Interrater agreement of VAS ratings among groups of raters for the three treatment groups, tested through the intraclass correlation coefficient (ICC; two-way mixed model, absolute agreement, results regarding average measures)

Interrater agreement	Face	Lower face	Upper lip	Lower lip	Chin
Activator	0.82 (0.59, 0.94)	0.81 (0.55, 0.94)	0.83 (0.61, 0.95)	0.79 (0.51, 0.93)	0.84 (0.63, 0.95)
Twin block	0.92 (0.80, 0.97)	0.93 (0.84, 0.98)	0.87 (0.70, 0.96)	0.83 (0.60, 0.95)	0.85 (0.61, 0.95)
Control group	0.89 (0.75, 0.97)	0.81 (0.55, 0.94)	0.81 (0.55, 0.94)	0.86 (0.67, 0.95)	0.85 (0.62, 0.95)

**Fig. 3:**
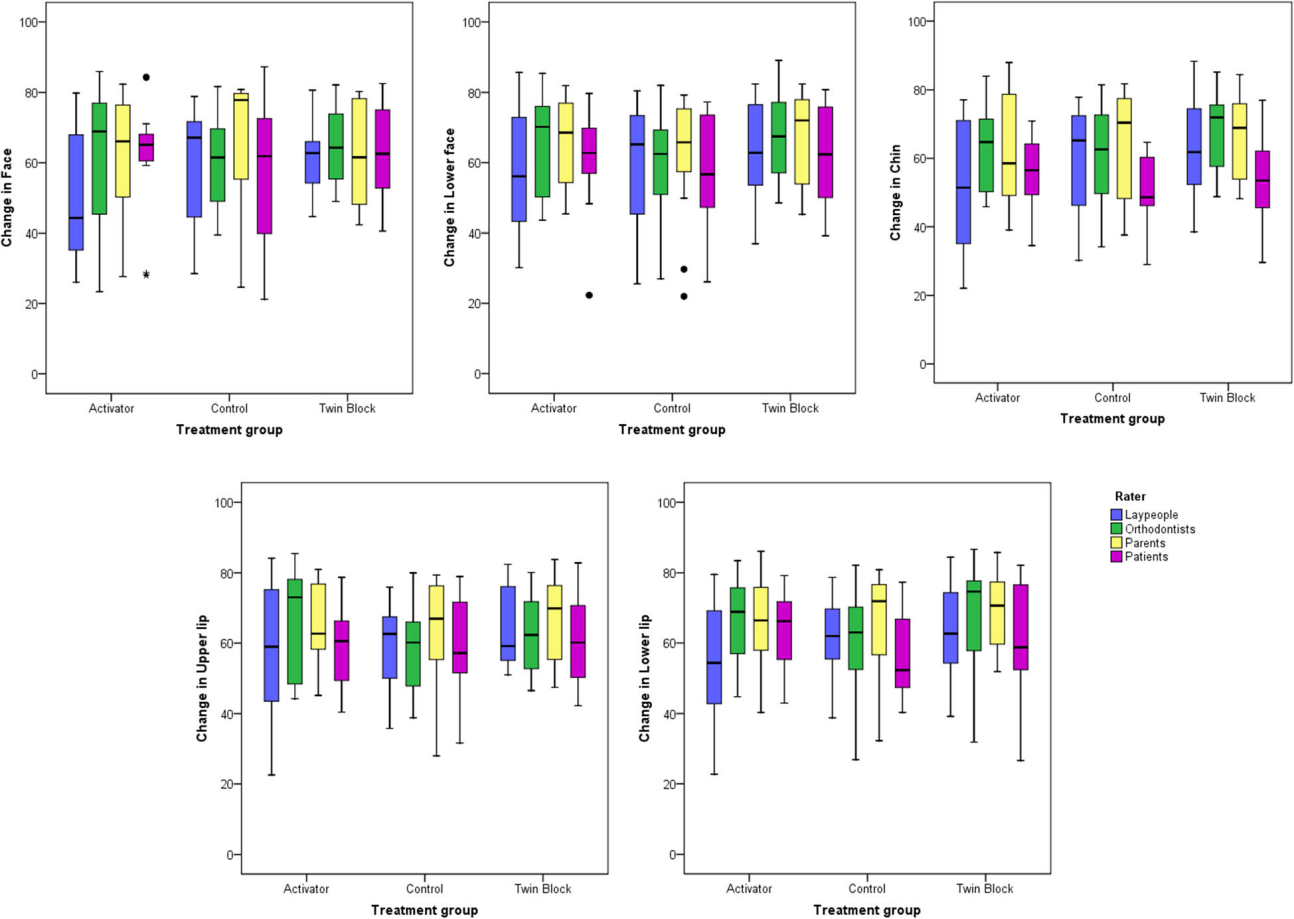
Box plots showing the assessed changes from pre- to post-treatment condition in VAS values (*y*-axis), grouped by treatment approach. The upper limit of the black line represents the maximum value, the lower limit the minimum value, the boxed the interquartile range, and the horizontal black line the median value. Outliers (> ± 3SD) are shown as black dots

**Fig. 4:**
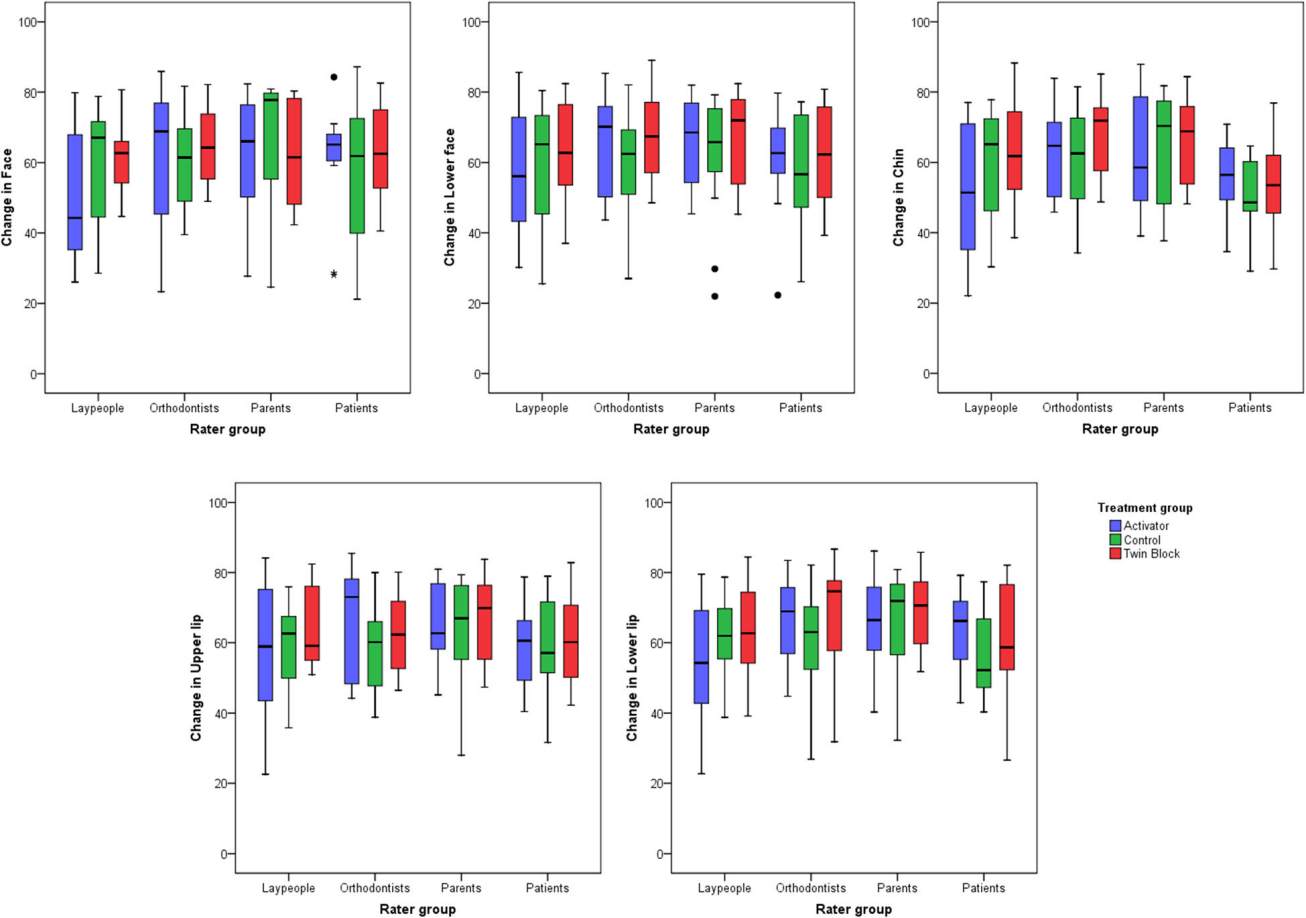
Box plots showing the assessed changes from pre- to post-treatment condition in VAS values (*y*-axis), grouped by rater type. The upper limit of the black line represents the maximum value, the lower limit the minimum value, the boxed the interquartile range, and the horizontal black line the median value. Outliers (> ± 3SD) are shown as black dots

Variances and covariances did not differ significantly between treatment groups (Levene’s test, *P* > 0.05). There were no significant differences among treatment groups (*F* = 0.91; *P* = 0.526; Wilks lambda = 0.93; partial *η*^2^ = 0.03) (Fig. 3), among raters (*F* = 1.68; *P* = 0.054; Wilks lambda = 0.83; partial *η*^2^ = 0.06) (Fig. 4), nor when testing the combined effect of the treatment group and rater on the results (*F* = 0.72; *P* = 0.866; Wilks lambda = 0.85; partial *η*^2^ = 0.03).

Raters assessed changes induced by aging and treatment as slightly positive in all treatment groups, although a wide individual variation was evident (Figs. 3 and 4). Tests of between-subjects effects did not reveal any significant differences among treatment groups (*P* > 0.05).

Although changes in the activator and twin-block groups were judged as slightly more positive than in the control group, particularly in the lower face and the lips, these findings were not statistically significant. Furthermore, their magnitude was negligible, since it hardly exceeded 1/20th of the total VAS length in its highest value (Table 5).

**Table 5:** Post hoc pairwise comparisons between treatment groups performed through Fisher’s least significant difference (LSD) test

Dependent variable	Treatment group (I)	Treatment group (J)	Mean difference (I–J)	SE	Sig.	95% confidence interval	
						Lower limit	Upper limit
Change in the face	Activator	Twin block	− 4.27	3.44	0.217	− 11.08	2.54
	Control	Activator	1.73	3.44	0.617	− 5.08	8.53
		Twin block	− 2.54	3.44	0.461	− 9.35	4.27
Change in the lower face	Activator	Twin block	− 3.41	3.15	0.280	− 9.64	2.81
	Control	Activator	− 2.39	3.15	0.449	− 8.62	3.83
		Twin block	− 5.81	3.15	0.067	− 12.03	0.42
Change in the upper lip	Activator	Twin block	− 1.77	2.81	0.530	− 7.33	3.79
	Control	Activator	− 2.12	2.81	0.452	− 7.68	3.44
		Twin block	− 3.89	2.81	0.169	− 9.45	1.67
Change in the lower lip	Activator	Twin block	− 3.19	2.88	0.269	− 8.88	2.50
	Control	Activator	− 1.59	2.88	0.582	− 7.28	4.10
		Twin block	− 4.78	2.88	0.099	− 10.47	0.91
Change in the chin	Activator	Twin block	− 4.23	2.95	0.154	− 10.07	1.61
	Control	Activator	0.10	2.95	0.973	− 5.74	5.94
		Twin block	− 4.13	2.95	0.164	− 9.97	1.70

*SE* standard error

## Discussion

Class II malocclusions, which have a common occurrence in contemporary societies, are reflected in the appearance of the lower face leading to a convex facial profile. This feature may negatively affect the esthetic appearance of the face. Thus, the improvement of facial convexity comprises a major aim of any such orthodontic treatment. The present study is the continuation of a previous one [9] evaluating the esthetic improvement of convex profile patients, after treatment with functional appliances followed by fixed orthodontic appliances. In that study, raters assessed facial profile photographs before and after the orthodontic intervention and perceived an improvement in the esthetic appearance of the face. On the contrary, the present study, where the raters assessed simultaneously frontal and profile photographs, did not reveal any significant treatment effect on patients’ facial appearance.

The methodology was identical to that of the previous study [9], enabling a direct comparison of the two, and allowing for an assessment of the potential influence that the addition of the frontal photograph has on evaluating orthodontic treatment outcomes. We found that any favorable treatment effects previously identified on facial profiles [9] diminished when a more global assessment of facial appearance was performed. This suggests that when the raters assessed only the profiles images, the treatment effect was evident, since the assessments focused exactly on the treatment target area, namely the facial profile. However, in the overall assessment of facial appearance, the raters probably did not only focus solely on the profile, but also on other facial features. These findings are in accordance with another study showing that facial convexity does not affect facial esthetic assessment of frontal photos at rest [20]. For proper interpretation of the findings, it should be considered that the objective profile improvement achieved in the present sample (Additional file 1: Table S1) is similar to the one reported in the literature for this treatment approach [5]. Furthermore, the overjet of the class II division 1 patients was considerably improved by treatment, reaching normal values, which also implies that the treatment was completed successfully.

Our findings add doubt to the premise that functional orthodontic treatment has a substantial favorable effect on patients’ facial appearance. Based on objective measurements, there was a definite improvement of the facial profile due to treatment and growth, though it did not reach control values; at T1, the median facial contour angle in the activator and twin-block groups was 17° (T0, 20°), whereas in the class I group, it was 12° (T0, 12°) (Additional file 1: Table S1). However, if this improvement is not perceivable by the human eye when the overall facial appearance is considered, then no positive effect of treatment on patients' lives is expected.

It is a common strategy in previous studies to use profile silhouettes, facial outlines, or black and white images in an attempt to control for confounding factors that may affect judgments of facial esthetics [16]. However, modified photos do not reflect the real conditions in everyday interactions and may also affect ratings inconsistently [9]. To our knowledge, both this and the previous study [9] are the only studies that used actual patient images to investigate the esthetic improvement of convex profile patients, after treatment with functional appliances followed by fixed orthodontic appliances. However, the present study design is a better simulation of actual human interaction, since people look at each other from various angels during social occurrences. It should be noted that both studies assessed the effect of treatment on static facial appearance, since that was the original aim. Facial expressions, such as the smile, may also influence the perception of facial esthetics [21]. Thus, a favorable effect of treatment in facial appearance during functioning cannot be excluded from the present findings. This might also be attributed to favorable changes in the dental appearance, which can then affect the overall facial appearance perception, though to a limited extent [22].

The intrarater error and the variation of assessments were higher in the present study compared to the previous one [9], suggesting that an increase of the given information added complexity to the way change was perceived by the human eye. However, in an actual everyday interaction, the information that the human eye transfers to the brain is quite higher, even compared to the present setup. Thus, the present design can be considered as more representative to actual conditions, compared to the previous one of profile assessment [9], but it still represents an oversimplification of the actual interactions between people in real-life conditions.

Subjective factors, such as personal opinion, environmental influence, ethnicity, and education, can affect the assessment of beauty and attractiveness by an individual [23, 24]. Experts may focus on achieving “flawless” skeletal and dentoalveolar class I relations, while laypersons may evaluate an individual’s appearance based on their personal experiences [25]. Therefore, the goals of orthodontic treatment set by professionals may not meet patients’ and parents’ expectations and may differ from laypersons’ assessments [3, 9, 18]. Nonetheless, orthodontic treatment should be able to improve a patient’s appearance in his/her eyes and in the eyes of laypeople, when such treatment goals have been set during planning.

A previous study on convex profile patients treated with surgical advancement of the mandible reported that a favorable treatment outcome was also seen on frontal photographs, though to a lesser degree compared to the profile assessments [24]. Thus, it could be argued that the changes induced by conventional orthodontic treatment did not reach certain thresholds, in regards to magnitude, to affect facial esthetic perception considerably. Furthermore, the same study found that the perceived improvement was doubled when the raters were aware of the treatment status, which is supporting our decision to not disclose this to the raters.

### Limitations

The most important limitation of the study is the retrospective collection of the rated cases. Retrospective studies are more susceptible to selection bias. To account for this, strictly defined eligibility criteria were applied to cases identified through a consecutive search of the archives. Thus, all patients that fulfilled these criteria were included, until the pre-determined sample size was reached. A further measure to minimize selection bias included the assessment of only the pre-treatment diagnostic records in the sample selection process. Thus, the risk to select cases based on the outcome was diminished. Post-treatment records were only used after the inclusion of a subject in the study. A full prospective randomized design would be ideal, but it might be unrealistic to be implemented due to time considerations.

The use of untreated class II division 1 patients could have also been an appropriate group to control for the effect of growth. We searched for such a group, but it was not possible to find one. It would have also been problematic to try to generate it, since not providing treatment to patients in need raises ethical and legal concerns. Even if available, this is not expected to have considerably affected the findings, since the effect of treatment and growth on a patient’s profile was found to be minimal, even in the treated group. The present control was suitable to test the effects of aging and setting factors, and thus, it met the needs of the study. The change of facial appearance perceived in the control group was mainly due to aging, since the profile was straight before and after treatment. In the convex profile group, more favorable change would have been seen if treatment had provided the desirable outcomes, those that are perceivable by people.

## Conclusions

The perceived facial changes of convex profile patients treated with functional appliances, followed by fixed orthodontic appliances, did not differ from those observed in normal profile patients, when full-face frontal and profile photos were simultaneously assessed. Consequently, professionals should be skeptical regarding the improvement of a patient’s facial appearance when this treatment option is used. Perhaps more drastic approaches should be considered in the case of convex profile patients with significantly compromised facial esthetics, especially when the patients’ and parents’ esthetic demands are high.

## Supplementary information

**Additional file 1: Table S1.** Sample characteristics.

## Data Availability

The datasets used and/or analyzed during the current study are available from the corresponding author on reasonable request.

## Abbreviations

ICC: Intraclass correlation coefficient
LSD: Least significant difference
SD: Standard deviation
VAS: Visual analog scale
