# Supplementary material for: Facial esthetic outcome of functional followed by fixed orthodontic treatment of class II division 1 patients
Source: Prog Orthod. 2019 Nov 25;20:42. doi: 10.1186/s40510-019-0294-9 (PMC6875534; doi:10.1186/s40510-019-0294-9)
Supplement: Supplementary file 1 — Additional file 1: Table S1. Sample characteristics. [file 40510_2019_294_MOESM1_ESM.docx]

**Table S1**. Sample characteristics.

| **Group** | **Sex** | **Age (yrs)**  Median (Range) | | **Treatment duration (yrs)**  Median (Range) | **Facial contour angle (**°**)**  Median (Range)* | | **Overjet (mm)**  Median (Range) | |
| --- | --- | --- | --- | --- | --- | --- | --- | --- |
|  |  | T0 | T1 |  | T0* | T1* | T0* | T1 |
| **Activator**  **(n = 12)** | 6M & 6F | 9.8 (9.2, 11.2) | 13.9 (12.8, 15.2) | 4.1 yrs (2.8, 5.0) | 20.5 (18.0, 28.0)^a^ | 17.0 (13.0, 22.5)^a^ | 8.0 (5.0, 12.0)^a^ | 2.0 (1.5, 3.0) |
| **Twin block**  **(n = 12)** | 6M & 6F | 10.6 (9.0, 11.9) | 13.5 (11.7, 16.7) | 3.6 yrs (1.6, 5.7) | 20.0 (17.0, 25.5)^a^ | 17.0 (10.5, 21.0)^a^ | 7.5 (5.0, 16.0)^a^ | 2.25 (1.5, 4.0) |
| **Control**  **(n = 12)** | 6M & 6F | 10.7 (8.9, 12.9) | 14.5 (13.1, 17.0) | 3.7 yrs (2.5, 5.9) | 12.3 (9.5, 14.5)^b^ | 12.0 (9.50, 15.0)^b^ | 3.5 (2.0, 5.0)^b^ | 2.0 (1.5, 2.5) |

M: males, F: females, yrs: years, T0: pre-treatment, T1: post-treatment

*Significant differences between the three groups (Kruskal-Wallis test, p < 0.05)

^a,b^Groups that did not differ significantly to each other are shown with similar superscript letters. Those that differ significantly to each other have dissimilar superscript letters (Mann-Whitney U test, p < 0.01)
